# Supplementary material for: The interactome of intact mitochondria by cross-linking mass spectrometry provides evidence for coexisting respiratory supercomplexes
Source: Mol Cell Proteomics. 2017 Dec 8;17(2):216–32. doi: 10.1074/mcp.RA117.000470 (PMC5795388; doi:10.1074/mcp.RA117.000470)
Supplement: Supplemental Data [file supp_RA117.000470_133922_0_supp_23978_fzffwf.zip › spectra_annotation/mito_spectra_annotation/152-1-7-1-7-1.pdf]

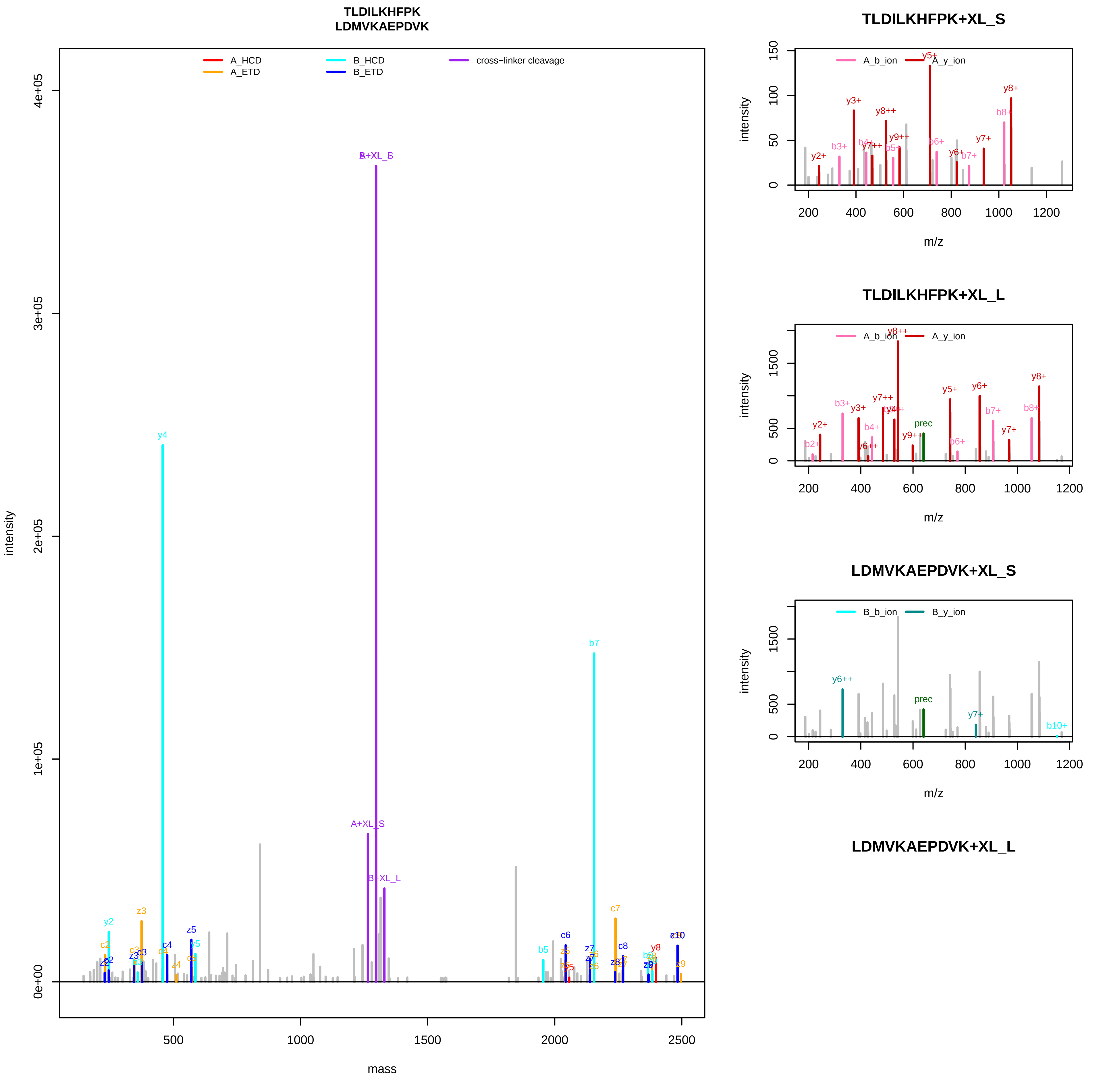

TLDILKHFPK
LDMVKAEPDVK
A_HCD
A_ETD
B_HCD
B_ETD
cross−linker cleavage
intensity
mass
4e+05
3e+05
2e+05
1e+05
0e+00
500
1000
1500
2000
2500
A+XL_S
B+XL_S
A+XL_S
B+XL_L
y4
b7
y2
z3
c7
y8
TLDILKHFPK+XL_S
A_b_ion
A_y_ion
m/z
TLDILKHFPK+XL_L
prec
LDMVKAEPDVK+XL_S
B_b_ion
B_y_ion
y6++
y7+
b10+
LDMVKAEPDVK+XL_L
